# Supplementary material for: Operation mode selection of NIMBY facility Public Private Partnership projects
Source: PLoS One. 2021 Jul 9;16(7):e0254046. doi: 10.1371/journal.pone.0254046 (PMC8270188; doi:10.1371/journal.pone.0254046)
Supplement: S1 Data — (PDF) [file pone.0254046.s001.pdf]

The information entropy data								
indicators	Expert 1	Expert 2	Expert 3	Expert 4	Expert 5	Expert 6	Expert 7	Expert 8
C1	0.0678	0.0809	0.0483	0.0759	0.0857	0.0759	0.0764	0.0086
C2	0.0313	0.0548	0.0396	0.0365	0.0000	0.0254	0.0000	0.0539
C3	0.0548	0.0183	0.0784	0.0744	0.0754	0.0571	0.0564	0.0485
C4	0.0705	0.0522	0.0532	0.0480	0.0698	0.0000	0.0877	0.0000
C5	0.0339	0.0574	0.0694	0.0617	0.0867	0.0481	0.0000	0.0476
C6	0.0496	0.1070	0.0577	0.0331	0.0377	0.0486	0.0466	0.0180
C7	0.0992	0.0000	0.0348	0.0682	0.0117	0.0288	0.0765	0.0613
C8	0.0392	0.0365	0.0264	0.0683	0.0587	0.0487	0.0576	0.0903
C9	0.0548	0.0615	0.0535	0.0491	0.0730	0.0309	0.0860	0.0717
C10	0.0261	0.6382	0.0299	0.0623	0.0465	0.0235	0.0754	0.0185
C11	0.0692	0.0000	0.0000	0.0465	0.0000	0.0662	0.0840	0.0703
C12	0.0454	0.0825	0.0151	0.0588	0.0186	0.0601	0.0281	0.0000
C13	0.0000	0.0428	0.0578	0.0000	0.0763	0.0526	0.0539	0.0653
C14	0.0574	0.0377	0.0000	0.0684	0.0343	0.0000	0.0566	0.0388
C15	0.0698	0.0293	0.0273	0.0437	0.0547	0.0648	0.0000	0.0378
C16	0.0388	0.0793	0.0846	0.0518	0.0365	0.0291	0.0462	0.0463
C17	0.0000	0.0364	0.0573	0.0253	0.0354	0.0372	0.0895	0.0723
