# Supplementary material for: Operation mode selection of NIMBY facility Public Private Partnership projects
Source: PLoS One. 2021 Jul 9;16(7):e0254046. doi: 10.1371/journal.pone.0254046 (PMC8270188; doi:10.1371/journal.pone.0254046)
Supplement: Attachment — Submitted filename: Response to Reviewers.docx [file pone.0254046.s002.docx]

**Response to Reviewer 1’s Comments**

First of all, thank you very much for your encouraging and inspiring feedback on my work and for your constructive and helpful comments that have definitely improved the paper a great deal. I had studied all of your comments carefully and tried to incorporate all of them into the current version of this article. Please find more detailed descriptions of how I did that below. We have modified our research to accommodate your concerns (Among them, the parts marked red in the manuscript are deletion, and the parts marked yellow are addition.). In addition, we provided a point-by-point response to each of your comment. For ease of distinction, your originals comments are listed below in regular font, and our responses to those comments are shown in italics font.

**1**.Please take care to the formulations. For example, the weight of a method does not sound well. Also, there are too many abbreviations.

**Response:**

*Thank you very much for your valuable comments and suggestions. The precedent version of the title has been replaced, becoming Operation mode selection of NIMBY facility Public Private Partnership projects.*

**2**. The abstract should be improved. It needs to cover five main elements: introduction, problem statement, methodology, contributions and results.

**Response:**

*Thank you for the suggestion. After reading the abstract carefully, we found that there was a lack of contributions and results. What we added is as follows* *(Lines 33-39, page 2). In this paper, combined weighting and TOPSIS method are applied to the research of NIMBY facility PPP project, and the operation mode selection of NIMBY facility PPP project is established, which makes up the blank of this part. Finally, a PPP project in Qingdao, Shandong Province, China, is taken as an example to verify the applicability of the model. The effectiveness of this model was tested by comparing the results of TOPSIS method, Grey target model, Extended matter-element mode and GRA-TOPSIS. It is hoped to provide useful reference for the operation mode selection of NIMBY facility PPP project.*

**3**. In introduction section the authors should provide more information about the existing models (GRA-TOPSIS and Entropy) used in the field and their benefits/weaknesses.

**Response:**

*Thanks for the reminder. We found that this section really needed to be added. Here's what we added:* *(Lines* *93-128, page 5-7) In the existing literature, no scholars have applied TOPSIS method and information entropy to the PPP project of NIMBY facilities, but there have been relatively mature studies in other fields. Zolfani et.al [14] used VIKOR and TOPSIS, two famous multi-attribute decisions making methods, to improve MADM. And the research study is focused on the analysis of the classical MADM methods based on logarithmic normalization. Durmić E et.al [15] combined Fucom method and Rough Saw model to solve the problem of operation mode selection, which provides a reference for the study of this paper. Dragan S et.al [16] proposed the BWM (Best Worst Method) and the COPRAS (Compressed Proportional Assessment) models for the selection of the optimal off-road vehicle for the needs of the SAF. Ramakrishnan et.al [17] solves the problem of green supplier selection in the automotive industry by combining the cloud model with TOPSIS technology. Mohammad Noureddine et.al [18] applied TOPSIS and MABAC (Multi-Attempted Border Approximation Comparison) to select the transport of Hazardous Material. Ivan Petrovic et.al [19] has determined and evaluated the selection criteria for the radar position of ATC, and the hybridized dematel-ahp-topsis model was modified by using the interval type-2 Fuzzy sets (IT2FS), to ensure the radar in air traffic management to play a successful role. Xingle. Teng et.al [20] through information entropy, grey correlation analysis method and TOPSIS method to determine the weight of indicators, judge the correlation degree and rank the decision units, and finally carry out a comprehensive evaluation of the development level of low-carbon economy in Shandong Province. Pengyu Dong et.al [21] combined the grey relational analysis (GRA) and TOPSIS method, and used the method of AHP and information entropy to put forward a GRA-TOPSIS radiation source threat assessment model based on game theory. Hedrea, EL et.al [22] presents the application of the tensor product (TP)-based model transformation approach to produce Tower CRrane (TCR) systems models. Through the analysis of previous scholars' articles, we can know that: GRA is a decision-making method to analyze "poor information" from a systematic point of view, which just overcomes the problems existing in the decision-making of TOPSIS method. GRA needs to determine the optimal value of each indicator, which is too subjective, and it is difficult to determine the optimal value of some indicators. TOPSIS method requires quantitative data, which are difficult and can only be used with more than two research objects. Therefore, the joint evaluation model of GRA and TOPSIS can not only consider the actual distance of the evaluated object in the multi-dimensional space, but also fully consider the correlation degree among various indicators. Entropy of information is a good description of the degree of chaos in a system, but it fails if the index changes very little or suddenly becomes larger or smaller.*

**4**. Why the grey correlation theory has been used in the TOPSIS method? What are the benefits of its implementation in TOPSIS?

**Response:**

*Thanks for your insightful comment. We have added this section in the article. (Lines 121-128, page 6-7) GRA is a decision-making method to analyze "poor information" from a systematic point of view, which just overcomes the problems existing in the decision-making of TOPSIS method. GRA needs to determine the optimal value of each indicator, which is too subjective, and it is difficult to determine the optimal value of some indicators. TOPSIS method requires quantitative data, which are difficult and can only be used with more than two research objects. Therefore, the joint evaluation model of GRA and TOPSIS can not only consider the actual distance of the evaluated object in the multi-dimensional space, but also fully consider the correlation degree among various indicators.*

**5**. Recent references in this field of work should be addressed in the overview:

A VIKOR and TOPSIS focused reanalysis of the MADM methods based on logarithmic normalization, Facta Universitatis series: Mechanical Engineering, 18(3), 341-355, 2020.

COPRAS model, Military Technical Courier, 68(1), 28-64, 2020.

Sustainable supplier selection using combined FUCOM - Rough SAW model, Reports in Mechanical Engineering, 1(1), 34-43, 2020.

Tensor product-based model transformation approach to tower crane systems modeling, Asian Journal of Control, DOI: 10.1002/asjc.2494, 2021

**Response:**

*We have read these articles carefully, and there are indeed many places worth learning from. The following parts are added:* *(Lines 96-103,116-118, page 4-6) Zolfani et.al [14] used VIKOR and TOPSIS, two famous multi-attribute decision making methods, to improve MADM. And the research study is focused on the analysis of the classical MADM methods based on logarithmic normalization. Durmić E et.al [15] combined Fucom method and Rough Saw model to solve the problem of operation mode selection, which provides a reference for the study of this paper. Dragan S et.al [16] proposed the BWM (Best Worst Method) and the COPRAS (Compressed Proportional Assessment) models for the selection of the optimal off-road vehicle for the needs of the SAF.* *Hedrea, EL et.al [22] presents the application of the tensor product (TP)-based model transformation approach to produce Tower CRrane (TCR) systems models.*

**6**. You should add step by step calculations in the case study section. You should also follow the steps defined in the preliminaries section.

**Response:**

*Thanks deeply for the suggestion. Because of the length, the steps in this part are indeed reduced, which is really not conducive to readers. We add the steps as follows (**Lines 554-571, page 30-31):*

*Step 1. Rank and score the indicators according to their importance*

$\boldsymbol{r}_{\boldsymbol{j}}$*=1.8,1.8,1.4,1.4,1.6,1.2,1.4,1.2,1.4,1.2,1.4,1,1.2,1.4,1.2,1.2,1.*

*Step 2. Subjective weight can be calculated according to Formula (1) and (2)*

$\omega_{J}^{'}$*=0.037,0.059,0.112,0.052,0.047,0.062,0.048,0.033,0.048,0.051,0.094,0.079,0.034,0.058,0.081,0.074,0.063.*

*Step 3. The information entropy is obtained by standardizing the data*

$E_{j}$*=0.9146,0.9697,0.8720,0.8313,0.7616,0.7152,0.7428,0.8530,0.8243,0.8172,0.8276,0.8749,0.8392,0.9426,0.8263,0.9163,0.8482.*

*Step 4. The objective weight is obtained by formula (7)*

$\omega_{j}^{"}$*=0.045,0.047,0.071,0.059,0.038,0.057,0.060,0.038,0.051,0.058,0.084,0.087,0.050,0.053,0.072,0.078,0.076.*

*Step 5. Substitute* $\omega_{J}^{'}$ *and* $\omega_{j}^{"}$ *into formula (9) and (10)*

$v_{1}^{'}$ *=0.42,*$v_{2}^{'}$*=-0.84*

*Step 6. Substitute* $v_{1}^{'}$*and* $v_{2}^{'}$*into formula (11)*

$v_{1}$*=0.5,*$v_{2}$*=0.5*

*Step 7. Substitute* $v_{1}$ *and* $v_{2}$ *into formula (8)*

$w_{j}$*=0.041,0.047,0.071,0.059,0.038,0.057,0.060,0.038,0.051,0.058,0.084,0.087,0.050,0.053,0.072,0.078,0.076.*

**7**. A validation section is missing. How can we assess and interpret the results? Comparison with the existing methods from the literature is missing.

**Response:**

*Thanks for your insightful comment. We have also taken into account the verification part, which can only be briefly mentioned due to the space**. (Lines 606-641, page 34-36)*

*In order to verify the rationality and accuracy of the method proposed in this paper, the single TOPSIS method, Grey target model [47] and the matter-element model [48] were respectively used to reorder the operation methods in the calculation examples in this paper, and the results were compared and analyzed. When selecting the evaluator, in order to ensure the reliability of the evaluation information, 8 experts in this field are still selected (Table 2) to evaluate the operating modes of the other 3 methods. After calculation, the results of the four methods are compared, and the results of different methods are listed in Table 7.*

*As can be seen from Table 7, the single TOPSIS method cannot better distinguish the differences and sensitivity of various schemes, leading to deviation in the results. However, the GRA-TOPSIS model can avoid this shortcoming, indicating that this method is more accurate and reasonable. GRA-TOPSIS method makes up for the shortcoming that TOPSIS method can only express the lack of distance and cannot determine the development trend of the scheme. It uses the grey correlation degree between the selected object and the positive and negative ideal solutions to construct the relative closeness degree to select the scheme, thus improving the effectiveness and scientific nature of the selection and making the relative closeness degree of the selection result more accurate. This will help decision makers make more accurate choices. However, GRA-TOPSIS method is not suitable for projects requiring small errors due to its low prediction accuracy.*

*The grey target model, the matter-element model and the GRA-TOPSIS model have the same calculation results, BOT is the best choice, but compared with the grey target model and the matter-element model, the GRA-TOPSIS model optimizes the calculation method of relative proximity degree, and its algorithm process is simpler, more operable and practical.*

*Through the above analysis we can see that in the adjacent from the infrastructure PPP project works in the process of selection, large extent the result of the choice of indicators are: C11 investment income level, the C3 project demand, C13 investment payback period, the C4 government PPP inherent factors such as experience, therefore, project earnings, project their own characteristics and the government capability of the adjacent facilities the choice of the PPP project works play a key role. The BOT method for financing the PPP project of NIMBY facilities can greatly relieve the financial pressure on the government, improve the operational efficiency of the PPP project of NIMBY facilities, reduce the financing risk, and thus obtain more comprehensive benefits.*

**8**. The resolution of figures requires improvement.

**Response:**

*Thanks for the suggestion. We have modified and uploaded it according to the format required by the journal.*

Finally, thanks deeply for your comments!

**Response to Reviewer 2’s Comments**

Thank you very much for your valuable comments and suggestions. We have modified our paper to accommodate your concerns (major changes in the article are highlighted in yellow for your quick perusal and the parts marked red in the manuscript are deletion. ). In addition, we provided a point-by-point response to each of your comment. For ease of distinct, your original comments are listed below in regular font, and our responses to those comments are shown in italics font.

**1**. The introduction section must be improved. Authors should better highlight the objective of their work and what its contributions are in order to close a gap to the existing literature and/or practice. What is the innovative value of the contribution proposed by the authors?

**Response:**

*Thanks for your suggestion. To make up for the lack of creativity and contribution and to make this article more logically rigorous, we have added the following (Lines 158-162, page 8): Fourth, the information entropy and GRA-TOPSIS methods have been very mature in other fields, but the research on the PPP project of NIMBY facilities by these methods is still blank. In this paper, information entropy and GRA-TOPSIS are applied for the first time to research on the operation mode of NIMBY facilities PPP project, making up the gap in this respect.*

**2**. In the introduction section, the authors should provide more information about existing MCDM models used in the field and their benefits/weaknesses.

**Response:**

*Thanks for the reminder. We found that this section really needed to be added. Here's what we added* *(Lines 93-128, page 5-7)*

*In the existing literature, no scholars have applied TOPSIS method and information entropy to the PPP project of NIMBY facilities, but there have been relatively mature studies in other fields. Zolfani et.al [14] used VIKOR and TOPSIS, two famous multi-attribute decisions making methods, to improve MADM. And the research study is focused on the analysis of the classical MADM methods based on logarithmic normalization. Durmić E et.al [15] combined Fucom method and Rough Saw model to solve the problem of operation mode selection, which provides a reference for the study of this paper. Dragan S et.al [16] proposed the BWM (Best Worst Method) and the COPRAS (Compressed Proportional Assessment) models for the selection of the optimal off-road vehicle for the needs of the SAF. Ramakrishnan et.al [17] solves the problem of green supplier selection in the automotive industry by combining the cloud model with TOPSIS technology. Mohammad Noureddine et.al [18] applied TOPSIS and MABAC (Multi-Attempted Border Approximation Comparison) to select the transport of Hazardous Material. Ivan Petrovic et.al [19] has determined and evaluated the selection criteria for the radar position of ATC, and the hybridized dematel-ahp-topsis model was modified by using the interval type-2 Fuzzy sets (IT2FS), to ensure the radar in air traffic management to play a successful role. Xingle. Teng et.al [20] through information entropy, grey correlation analysis method and TOPSIS method to determine the weight of indicators, judge the correlation degree and rank the decision units, and finally carry out a comprehensive evaluation of the development level of low-carbon economy in Shandong Province. Pengyu Dong et.al [21] combined the grey relational analysis (GRA) and TOPSIS method, and used the method of AHP and information entropy to put forward a GRA-TOPSIS radiation source threat assessment model based on game theory. Hedrea, EL et.al [22] presents the application of the tensor product (TP)-based model transformation approach to produce Tower CRrane (TCR) systems models. Through the analysis of previous scholars' articles, we can know that: GRA is a decision-making method to analyze "poor information" from a systematic point of view, which just overcomes the problems existing in the decision-making of TOPSIS method. GRA needs to determine the optimal value of each indicator, which is too subjective, and it is difficult to determine the optimal value of some indicators. TOPSIS method requires quantitative data, which are difficult and can only be used with more than two research objects. Therefore, the joint evaluation model of GRA and TOPSIS can not only consider the actual distance of the evaluated object in the multi-dimensional space, but also fully consider the correlation degree among various indicators. Entropy of information is a good description of the degree of chaos in a system, but it fails if the index changes very little or suddenly becomes larger or smaller.*

**3**. Why the TOPSIS method? Why not RAFSI, MABAC, MAIRCA, VIKOR methods? This should be discussed. The authors need to discuss their contributions compared to those methods in related papers.

**Response:**

*Thanks for your suggestion. The above methods should be discussed in the study of multi-attribute decision making problem. We add them as follows (Lines 78-91, page 4-5):*

*Common multi-attribute decision making methods include RAFSI, MABAC, MAIRCA, VIKOR, etc. RAFSI(Ranking of Alternatives through Functional mapping of criterion sub-intervals into a Single Interval) could rank the Alternatives through Functional mapping of criterion sub-intervals into a Single Interval, which successfully eliminates the rank reversal problems[9]. MABAC（multi-attributive border approximation area comparison）model, which handles the complex and uncertain decision making issues by computing the distance between each alternative and the bored approximation area（BAA）,has been investigated by an increasing number of researchers more recent years[10]. MAIRCA (multi attribute ideal real comparative analysis) can be used for quantitative and qualitative comprehensive analysis, with the advantages of objective evaluation, stable calculation results, and the calculation amount will not change greatly with the change of the number of variables [11]. Can VIKOR consider group utility maximization and individual regret minimization when decision-makers don't know how to express preferences[12]. The methods mentioned above can be used to build solutions and best ranking models.*

**4**. The authors must clearly discuss the significance of the research problem in the first section.

**Response:**

*Thanks for your insightful comment. In the abstract* *we added as following (Lines 33-39, page 2):*

*In this paper, combined weighting and TOPSIS method are applied to the research of NIMBY facility PPP project, and the operation mode selection of NIMBY facility PPP project is established, which makes up the blank of this part. Finally, a PPP project in Qingdao, Shandong Province, China, is taken as an example to verify the applicability of the model. The effectiveness of this model was tested by comparing the results of TOPSIS method, Grey target model, Extended matter-element mode and GRA-TOPSIS. It is hoped to provide useful reference for the operation mode selection of NIMBY facility PPP project.*

*In the Introduction, we have added as following (**Lines 158-162, page 8): Fourth, the information entropy and GRA-TOPSIS methods have been very mature in other fields, but the research on the PPP project of NIMBY facilities by these methods is still blank. In this paper, information entropy and GRA-TOPSIS are applied for the first time to the research on the operation mode of NIMBY facilities PPP project, making up the gap in this respect.*

**5**. The authors should explain why they insist on using the G1 subjective weight model. Why not LBWA, FUCOM or BWM models? These models have numerous advantages and the authors should show what the advantages of G1 over LBWA, FUCOM or BWM models are. These methods should be discussed in the paper.

**Response:**

*Thanks for the helpful suggestion. We added as following (Lines 360-370, page 20-21):*

*There are many commonly used subjective weighting methods, such as LBWA, FUCOM, BWM etc. The LBWA model is based on a pairwise comparison of the criteria through forming a non-decreasing array of the criteria important levels. It enables rationally expressing the judgments of a decision-maker with a minimum number of comparisons [51]. Ahmad Naim et.al [52] compared FUCOM and BWM and concluded that BWM has less pair-wise Verbs and leads to FUCOM more reliable solution. The author claimed that FUCOM offers better consistency, reduced pair-wise comparisons, and flexibility of the measurement scale. Different from the above methods, G1 method does not need consistency test and is more convenient to operate. Therefore, this paper chooses G1 method to calculate the weight.*

**6**. Why the Entropy method was used (objective weight model)? Why not CRITIC, FANMA, etc?

**Response:**

*Thanks for the reminder. The relevant content is shown as follows (Lines 374-384, page 21-22):*

*There are many commonly used objective weighting methods, such as CRITIC, FANMA, information entropy，etc. Milićević et.al [53] proposed the information entropy method, the CRITIC method and the Fanma method, and compared and analyzed the three methods. CRITIC considers the size of the variability of indicators while taking into account the correlation between indicators, not that the greater the number is, the more important it is, and makes full use of the objective attributes of the data itself for scientific evaluation. Fanma is objective and generates weight of criteria directly from the criterion value variants. It also eliminates the problem of subjectivity and incompetence. Compared with other objective weighting methods, the use of information entropy is not limited by its scope. It can be used in any evaluation problem, and it can also eliminate the indexes that do not contribute much to the evaluation results in the index system.*

**7**. The authors should provide more recent references published in the previous two-three years. References published prior to 2017 may be used, but those are generally of less interest. Some recent interesting references from the MCDM field are missing (application of TOPSIS method). I suggest to the authors to read and discuss the below listed references:

A VIKOR and TOPSIS focused reanalysis of the MADM methods based on logarithmic normalization. Facta universitatis series: Mechanical Engineering, 18(3), 341-355.;

A cloud TOPSIS model for green supplier selection. Facta Universitatis, series: Mechanical Engineering. 18(3), pp. 375-397.;

Route planning for hazardous materials transportation: Multicriteria decision making approach. Decision Making: Applications in Management and Engineering, 2(1), 66-85.

A hybridized IT2FS-DEMATEL-AHP-TOPSIS multicriteria decision making approach: Case study of selection and evaluation of criteria for determination of air traffic control radar position. Decision Making: Applications in Management and Engineering, 3(1), 146-164.

**Response:**

*Thanks deeply for the suggestion. We have read the above articles carefully and added them as follows (Lines 96-101,103-109 page 5-6):*

*Zolfani et.al [14] used VIKOR and TOPSIS, two famous multi-attribute decisions making methods, to improve MADM. And the research study is focused on the analysis of the classical MADM methods based on logarithmic normalization. Ramakrishnan et.al [17] solves the problem of green supplier selection in the automotive industry by combining the cloud model with TOPSIS technology. Mohammad Noureddine et.al [18] applied TOPSIS and MABAC (Multi-Attempted Border Approximation Comparison) to select the transport of Hazardous Material. Ivan Petrovic et.al [19] has determined and evaluated the selection criteria for the radar position of ATC, and the hybridized dematel-ahp-topsis model was modified by using the interval type-2 Fuzzy sets (IT2FS), to ensure the radar in air traffic management to play a successful role.*

**8**. The authors should provide step by step calculations for suggested methodology, especially Comprehensive weight model. The methodology should be explained in details.

**Response:**

*Thanks deeply for the suggestion. Because of the length, the steps in this part are indeed reduced, which is really not conducive to readers. We add the steps as follows: (**Lines 554-571, page 30-31)*

*Step 1. Rank and score the indicators according to their importance*

$\boldsymbol{r}_{\boldsymbol{j}}$*=1.8,1.8,1.4,1.4,1.6,1.2,1.4,1.2,1.4,1.2,1.4,1,1.2,1.4,1.2,1.2,1.*

*Step 2. Subjective weight can be calculated according to Formula (1) and (2)*

$\omega_{J}^{'}$*=0.037,0.059,0.112,0.052,0.047,0.062,0.048,0.033,0.048,0.051,0.094,0.079,0.034,0.058,0.081,0.074,0.063.*

*Step 3. The information entropy is obtained by standardizing the data*

$E_{j}$*=0.9146,0.9697,0.8720,0.8313,0.7616,0.7152,0.7428,0.8530,0.8243,0.8172,0.8276,0.8749,0.8392,0.9426,0.8263,0.9163,0.8482.*

*Step 4. The objective weight is obtained by formula (7)*

$\omega_{j}^{"}$*=0.045,0.047,0.071,0.059,0.038,0.057,0.060,0.038,0.051,0.058,0.084,0.087,0.050,0.053,0.072,0.078,0.076.*

*Step 5. Substitute*$\omega_{J}^{'}$ *and* $\omega_{j}^{"}$ *into formula (9) and (10)*

$v_{1}^{'}$*=0.42,*$v_{2}^{'}$*=-0.84*

*Step 6. Substitute* $v_{1}^{'}$*and* $v_{2}^{'}$*into formula (11)*

$v_{1}$*=0.5,*$v_{2}$*=0.5*

*Step 7. Substitute* $v_{1}$ *and* $v_{2}$ *into formula (8)*

$w_{j}$*=0.041,0.047,0.071,0.059,0.038,0.057,0.060,0.038,0.051,0.058,0.084,0.087,0.050,0.053,0.072,0.078,0.076.*

**9**. How can a reader judge about the quality of the obtained solutions? Could the results be compared with some existing approaches in literature? The improvement must be discussed. Suggestion: compare the obtained results with results yielded by the classic TOPSIS method and, in doing that, compare the advantages and limitations of your model over the classic TOPSIS method.

**Response:**

*Thanks deeply for the suggestion. The effectiveness of the model was tested by comparing the results of TOPSIS method, Grey target model, Extended matter-element mode and GRA-TOPSIS**, (Lines 606-619, page 34-35). We add the advantages and disadvantages of this model as follows (**Lines 619-626, page 35): GRA-TOPSIS method makes up for the shortcoming that TOPSIS method can only express the lack of distance and cannot determine the development trend of the scheme. It uses the grey correlation degree between the selected object and the positive and negative ideal solutions to construct the relative closeness degree to select the scheme, thus improving the effectiveness and scientific nature of the selection and making the relative closeness degree of the selection result more accurate. This will help decision makers make more accurate choices. However, GRA-TOPSIS method is not suitable for projects requiring small errors due to its low prediction accuracy.*

**10**. A validation section is missing.

**Response:**

*Thanks for your insightful comment. We have also taken into account the verification part, which can only be briefly mentioned due to the space. (Lines 606-641 page 34-36)*

*In order to verify the rationality and accuracy of the method proposed in this paper, the single TOPSIS method, Grey target model [47] and the matter-element model [48] were respectively used to reorder the operation methods in the calculation examples in this paper, and the results were compared and analyzed. When selecting the evaluator, in order to ensure the reliability of the evaluation information, 8 experts in this field are still selected (Table 2) to evaluate the operating modes of the other 3 methods. After calculation, the results of the four methods are compared, and the results of different methods are listed in Table 7.*

*As can be seen from Table 7, the single TOPSIS method cannot better distinguish the differences and sensitivity of various schemes, leading to deviation in the results. However, the GRA-TOPSIS model can avoid this shortcoming, indicating that this method is more accurate and reasonable. GRA-TOPSIS method makes up for the shortcoming that TOPSIS method can only express the lack of distance and cannot determine the development trend of the scheme. It uses the grey correlation degree between the selected object and the positive and negative ideal solutions to construct the relative closeness degree to select the scheme, thus improving the effectiveness and scientific nature of the selection and making the relative closeness degree of the selection result more accurate. This will help decision makers make more accurate choices. However, GRA-TOPSIS method is not suitable for projects requiring small errors due to its low prediction accuracy.*

*The grey target model, the matter-element model and the GRA-TOPSIS model have the same calculation results, BOT is the best choice, but compared with the grey target model and the matter-element model, the GRA-TOPSIS model optimizes the calculation method of relative proximity degree, and its algorithm process is simpler, more operable and practical.*

*Through the above analysis we can see that in the adjacent from the infrastructure PPP project works in the process of selection, large extent the result of the choice of indicators are: C11 investment income level, the C3 project demand, C13 investment payback period, the C4 government PPP inherent factors such as experience, therefore, project earnings, project their own characteristics and the government capability of the adjacent facilities the choice of the PPP project works play a key role. The BOT method for financing the PPP project of NIMBY facilities can greatly relieve the financial pressure on the government, improve the operational efficiency of the PPP project of NIMBY facilities, reduce the financing risk, and thus obtain more comprehensive benefits.*

**11**. In the conclusion section, the authors need to demonstrate the impact and insights of the research. The authors need to clearly provide several solid future research directions and their research contributions. The limitations of the model should be added.

**Response:**

*Thanks for the important suggestion. What we added is as follows (Lines 666-670, page 37): The goals of future research should be aimed into the direction of using the GRA-TOPSIS method for other real problems as well as combining with objective and subjective criteria weighting techniques. Furthermore, one of the goals of future research also lies in expanding GRA-TOPSIS method by using different uncertainty theories.*

Finally, thanks deeply for your comments!
