# Supplementary material for: Operation mode selection of NIMBY facility Public Private Partnership projects
Source: PLoS One. 2021 Jul 9;16(7):e0254046. doi: 10.1371/journal.pone.0254046 (PMC8270188; doi:10.1371/journal.pone.0254046)
Supplement: Attachment — Submitted filename: Response to Reviewers.docx [file pone.0254046.s003.docx]

Response to Reviewer 1’s Comments

Thank you very much for your valuable comments and suggestions. We attach great importance to your suggestion on revising the reference. We have checked all the references and highlighted the changes in yellow. Thank you again for your help.

Response to Reviewer 2’s Comments

First of all, thank you very much for your encouraging and inspiring feedback on my work and for your constructive and helpful comments that have definitely improved the paper a great deal. We attach great importance to your suggestion on revising the reference. We have checked all the references and highlighted the changes in yellow. Thank you again for your help.
